# Employee-Athletes: Exploring the Elite Spanish Athletes' Perceptions of Combining Sport and Work

**DOI:** 10.3389/fpsyg.2021.633133

**Published:** 2021-02-23

**Authors:** Rubén Moreno, José L. Chamorro, Cristina López de Subijana

**Affiliations:** ^1^Department of Sports Sciences, Faculty of Sport Sciences, Universidad Europea de Madrid, Madrid, Spain; ^2^Social Sciences Applied to Sport, Physical Activity and Leisure Department, Universidad Politécnica de Madrid, Madrid, Spain

**Keywords:** dual career, qualitative, resources, barriers, elite athletes, employee-athletes

## Abstract

Researchers have studied the athletes' dual careers with the aim of helping them to combine the sport and the academic–vocational sphere. Most of this research has addressed the study–sport combination, but there is a lack of studies on the work–sport combination. The main objective of this research was to explore the subjective perceptions of Spanish elite athletes when attempting to combine their careers as professional athletes with a second profession or trade. Further, this study aims to identify the access to facilitating resources and the perception of obstacles and barriers to the development of a dual career. A qualitative approach was chosen to address these research questions. Interpretative phenomenological analysis was carried out on a sample of 18 elite athletes, and the data were collected using semi-structured interviews based on a set of superordinate and subordinate categories. The results showed that the athletes interviewed possessed valuable resources such as the transference of sports values to the work sphere. However, important barriers were highlighted such as the perception of sport institutions as absent entities in the work–sport combination. Thus, the study of this type of dual career seems to be one of the challenges facing the interested scientific community for the coming years.

## Introduction

In the last two decades, studies in transitions and career development in sport have increased in both quantity and quality. Currently, the holistic view of the athlete predominates in the approach of this type of studies (Stambulova and Wylleman, 2019). As will be seen below, several studies and research projects have focused their efforts on investigating the role of dual careers (DCs) in athletic career development and transition. A DC can briefly be defined as “a career focused on sport and education or work” (Stambulova and Wylleman, 2015, p. 1). However, in the critical review of DC studies carried out by Stambulova and Wylleman (2019), only two studies out of 42 considered some aspects of DCs in sport and work. With the purpose of contributing to the increase of knowledge about this topic, the present work explores the Spanish elite athletes' perceptions of combining sport and work.

According to Schlossberg (1981) definition, transitions can be described as “an event or non-event which results in a change in assumptions about oneself and the world and thus requires a corresponding change in behaviors and relationships” (p. 5). In light of this, the transition to retirement from elite sport should be understood as a process that occurs over time, and not as a specific and limited event (Barker et al., 2014). The first studies on transitions related to professional sport, dating back to the 1970's, were focused on the negative consequences of retirement (Park et al., 2013). Only toward the end of the twentieth century were transitional models applied from a general perspective (e.g., Bloom, 1985; Salmela, 1994; Stambulova, 1994; Wylleman et al., 2004). Since then, sports psychologists have attempted to protect the interests of young athletes and, in particular, their educational and vocational rights, in order to promote their social and professional integration in the labor market after their athletic careers (Capranica and Millard-Stafford, 2011; European Commision, 2012). To this purpose, many of them followed the assumption that higher education generally improves the chances of employment, since the labor market has become particularly difficult to access in times of crisis (2008–2018), favoring those possessing a university degree (Organisation for Economic Cooperation Development, 2017).

Different models have addressed athletic career development and transitions to provide theoretical support to scientists and practitioners. The sport career transition model (Stambulova, 2003) proposes that the athletic career transition is perceived as a process of coping with a set of specific demands/challenges that must be faced for continuing an athletic career successfully or adjusting to the post-sport career. Effective coping will depend on the balance between resources (factors that facilitate the coping process) and barriers (factors that interfere with effective coping) that athletes have available.

In addition, Wylleman and Lavalle (2004) developed a holistic model that assumes the interactivity and interdependence of sport career transitions, which crystallized into the holistic athletic career model (HACM; Wylleman et al., 2013; Wylleman, 2019). This model reflects (a) the concurrent, interactive, and reciprocal nature of the athlete's development across six dimensions (sporting, psychological, psychosocial, academic–vocational, financial, and legal); (b) the normative transitions at each of the four developmental dimensions (initiation, development, mastery, and discontinuation or retirement); and (c) an approach focused on the concept of “global career,” as well as a perspective based on the development of the “person as a whole,” extending across several different aspects. This interaction between dimensions and stages makes the model dynamic, which is a differentiating element compared with others (Wylleman et al., 2013; Torregrosa et al., 2016), facilitating study of this complex reality and representing one of the dominant theoretical frameworks of the last few years (Stambulova and Wylleman, 2019).

Both models have provided a theoretical framework that scientists could follow for their research, identifying the stages of the athletic career with the transitions, the different dimensions and life spheres, and factors that facilitate or hinder coping (Torregrosa et al., 2020). Regarding some key factors affecting successful transitions out of competitive sport, several studies recommend that the athlete should plan their retirement, should be able to maintain a wide social network inside and outside of sport, should not exclusively develop a sports identity and perceive the transition as a process, and, lastly, should maintain a global vision on the extension of the sports career from a vital perspective lasting longer than the 10 years at the high level (Torregrosa et al., 2004, 2015; Pallarés et al., 2011; Park et al., 2013; Barker et al., 2014; Tshube and Feltz, 2015; Jordana et al., 2017; Lupo et al., 2017; Carapinheira et al., 2018; Monteiro et al., 2020). Therefore, career assistance for athletes is based on a number of principles such as using a global and multilevel approach (Alfermann and Stambulova, 2007).

Furthermore, the holistic vision of athletic career development proposed by the HACM suggests that athletic and academic/vocational levels could interact with each other. In European discourse, the term to identify careers focusing on sport and education or work has been identified as DCs on the sport, and the education or work has been identified as DC (Stambulova and Wylleman, 2015). In this context, some researchers have focused on generating programs aimed at helping young athletes to combine their careers in sports with their academic or professional development, leading to less traumatic transitions after their retirement (Stambulova et al., 2009, 2020). Besides, these considerations gain even more relevance when considering the results of the Labor Force Survey administered by the Instituto Nacional de Estadística (INE; National Institute of Statistics), in collaboration with Eurostat (Instituto Nacional de Estadística, 2016). According to this research, 41.68% of the Spanish population between 16 and 34 years find their first employment through their social network, whereas only 2.4% were successfully employed through job centers or other government agencies. This may emphasize the need to broaden the spectrum of socialization that should be inherent in the pursuit of a DC.

Since the beginning of the twenty first century, in Europe, there seems to be increased interest in DC research (Guidotti et al., 2015; Stambulova and Wylleman, 2019). Some studies underlined how the difficulties experienced in combining professional sport and education could lead to personal discomfort, poor academic results, stress, loss of motivation, mental illness, and the perception of a negative impact of sports careers on academic performance (Christensen and Sørensen, 2009; Enoksen, 2011). However, other studies suggested that athletes who combine sport and education achieved good academic results and reported high satisfaction levels if they studied in a center that supported both the academic and athletic career (Stambulova and Ryba, 2014; Stambulova et al., 2014; Wartenberg et al., 2014). This is of great value because the duration and the quality of the education received have an important impact on the transition from the education to labor market in the general population (Organisation for Economic Cooperation Development, 2017). According to this same source, the transition is an important societal concern, because young people stay longer in the education system in order to improve their skills while they wait until the economic and work conditions improve.

Recently, Stambulova and Wylleman (2019) showed in a review of studies on DC between 2015 and 2018 that the vast majority of papers addressed the combination of sport and education, and they recommend an increase in the research on DC in sport and work. This recommendation follows a change in the political trend, since, already in 2009, new policies were being implemented in relation to the employment, education, and enabling of elite athletes (Guidotti et al., 2015). These policies led to several projects aimed at the smooth development of the DC. However, only one of these initiatives, namely, an Erasmus+ project, has been launched to specifically address the combination of academic career, professional development, and sport. The project—“Be a Winner in elite Sport and Employment before and after Athletic Retirement” (B-WISER, 2018)—reveals a shift in perspective insofar as focuses on studying employability skills in active and retired athletes and on access to the labor market from the employer's perspective (Stambulova and Wylleman, 2019; Torregrosa et al., 2020).

The scarcity of studies investigating DC focusing on the relationship between the sports career and employment has been attributed by some to the lack of support for working athletes (Debois et al., 2015) or to the fact that women are better than men at combining work and sport, particularly highly specialized sport (Chamorro et al., 2015; Ryba et al., 2015; Ekengren et al., 2018). In any case, the overwhelming majority of the studies on DC are concerned with the relationship between sport and education (Stambulova et al., 2014; Miró et al., 2018; Moreno et al., 2018; Sánchez-Pato et al., 2018). Keeping this general trend in mind, more studies investigating DC among working athletes are needed to lead to a better understanding of the phenomenon as a whole. Thus, the objective of the present research was to explore the subjective perception of the resources and barriers that employee-athletes have to face in their DC process.

## Materials and Methods

### Participants

The intentional sample was formed by 18 Spanish elite athletes (medalists in European and World Championships and/or Olympic Games) and was carried out during the first 6 months of 2018. Two inclusion criteria were used for participant selection: (a) being included in the national government list of elite athletes and (b) having won at least one medal in European, World, or Olympic championships. And one exclusion criterion was used: (a) being an athlete in a highly professional sport (e.g., football or basketball). Ten were retired, and eight were still active. All of them worked during their sports career. The sample was composed of 10 men and eight women between 20 and 65 years old (M = 39.83; SD = 12.45). The nature of the jobs varied from skilled work (e.g., hired in functions that require higher studies, as well as self-employed jobs that may not require higher studies), to unskilled work (e.g., occasional jobs and irregular work that do not require higher studies). The athletes were randomly assigned a number from 1 to 18 to ensure anonymity (Table 1).

**Table 1:** Demographic details of athletes.

**Athletes**	**Sex**	**Sport**	**Work**	**Years of practice**	**Active/retired**
D1	Man	Individual	Unskilled	15	Retired
D2	Woman	Individual	Skilled	18	Retired
D3	Man	Individual	Unskilled	14	Retired
D4	Man	Individual	Skilled	20	Retired
D5	Man	Individual	Unskilled	19	Retired
D6	Woman	Individual	Skilled	12	Retired
D7	Woman	Team	Skilled	21	Retired
D8	Woman	Team	Unskilled	22	Retired
D9	Man	Team	Skilled	25	Retired
D10	Woman	Individual	Skilled	21	Retired
D11	Man	Team	Skilled	30	Active
D12	Woman	Team	Unskilled	13	Active
D13	Man	Individual	Skilled	17	Active
D14	Man	Individual	Skilled	16	Active
D15	Woman	Individual	Unskilled	15	Active
D16	Man	Team	Skilled	12	Active
D17	Man	Individual	Skilled	18	Active
D18	Woman	Individual	Unskilled	10	Active

### Instrument

In-depth interviews were conducted based on a semi-structured script focused on the psychological, psychosocial, academic–vocational and financial levels of the HACM (Wylleman et al., 2013). The script addressed topics like the athletes' personal experiences in relation to DC, their sources of support, their motivations in relation to work, or their sources of income, following the underlying model that worked as a lens to build our vision of participant's events in terms of career and developmental stages (Debois et al., 2015). Drawing on Patton (2002), this script consisted of flexible questions, so that the participants could describe significant facts from their sports and academic–vocational careers, granting a certain freedom to the interviewers and the interviewees.

### Procedure

The sample was selected after approval by the ethics committee of the institution that conducted the research. Personal contacts of the authors were selected using a “snowball sampling” technique (Baltar and Gorjup, 2012). This sampling is the one most commonly recommended when studying subjects belonging to elite groups (Finkel et al., 2008), as in the case with medalists in international championships. In the composition of the sample, an attempt was made to seek parity. For this reason, these groups were created to represent women and men, active and retired, to cover as wide a spectrum as possible. Later, the category of team sport or individual sport was added to further this idea. It is important to take into account how the quality of the sample could provide credibility and transferability to be considered representative of a larger population. Furthermore, data collection was carried out until saturation. In the field of qualitative research, saturation is understood as the point at which a certain diversity of ideas has already been heard and with each additional interview no other elements appear. Subjects were contacted by telephone, arranging a place of their liking for the interview. They all lived in Madrid. The date and time were arranged in accordance with the athlete's agenda. During the interviews, the athletes were informed about the purpose of the study, as well as the anonymous data processing, following the ethical requirements of the code of conduct of the American Psychological Association (2010). All participants signed an informed consent form before the interview, and they were informed of their right to leave at any moment if they felt it was necessary. The interviews lasted between 45 and 90 min and were recorded. The interviews were carried out following the recommendations of Smith and Sparkes (2017).

### Analysis of the Data

The recordings of the interviews were transcribed using *Verbatim* and were read several times for their further qualitative analysis through the Atlas.ti computer program version 7. This whole process was carried out in the Sports Faculty of the Universidad Europea de Madrid. Interpretative phenomenological analysis (IPA) is a qualitative methodology that aims to explore and understand the vision of each athlete, not to describe an objective reality like most research methodologies. Therefore, it is necessary to achieve an in-depth insight of the participants' perceptions, emotions, thoughts, and feelings. Then the quotes that better reflected personal experiences in relation to the research objective were identified and codified (Smith, 1996; Smith and Osborn, 2007)

Different categories were created with representative meanings in order to make sense of the material originating from the statements made by the informants and/or from descriptions of phenomena or processes provided by them (Gómez et al., 1999; Smith and Sparkes, 2017; Smith, 2018). Afterwards, these first categories were used to develop emerging themes that would condense the data and capture the essential characteristics of the narratives. These emerging themes were grouped depending on the central meaning or concept shared in order to develop supraordinate categories. The emerging categories, as well as the supraordinate ones, were evaluated following patterns, similarities, and differences, resulting in subordinate themes. In this way, it was possible to link the athletes' experiences with themes of a superior order, reflecting the differences, which is the central focus of IPA. Finally, an internal coherence analysis was carried out among the article's three authors, in order to increase precision and corroborate consistency (Thomas et al., 2015).

## Results

The results are presented based on the four levels of the model that supports this research: (1) psychological, (2) psychosocial, (3) academic–vocational, and (4) financial. In accordance with the proposed aim of the study, the supraordinate categories with representative meaning found were included in each level. In line with the objective proposed, the different subordinate themes were assigned to the “resource” or “barrier” categories. Table 2 shows the main results.

**Table 2:** Resources and barriers perceived by the employee-athletes in their dual careers.

**Levels**	**Psychological**		**Psychosocial**		**Academic–vocational**		**Financial**	
**Supraordinate categories**	**Sports identity**		**Social support perceived**		**Work–sport combination**		**Security perception**	
**Resources barriers**	**Resource**	**Barrier**	**Resource**	**Barrier**	**Resource**	**Barrier**	**Resource**	**Barrier**
Subordinate categories	Transference of sports values to the labor sphere (18)	Effort and requirement perception (18)	Family role (8)	Social network (11)	Coping strategies for retirement (18)	Obstacles in positions within companies not dedicated to sports (6)	Income (8)	Income (10)
Subordinate categories	Underlying sports identity (11)				Facility for the work–sport combination (4)			Perception about the sports entities (16)
Subordinate categories					Facility for the combination within the private labor sector (8)			

*In parentheses is the number of athletes who fits in the general results obtained*.

### Psychological Level

The psychological level describes each athlete's individual experiences through their DC. The emerging supraordinate category was “sports identity.”

#### Resources

##### Transference of Sports Values to the Labor Sphere

The 18 participants reported that the transference of sports values to the labor sphere was crucial to be able to properly execute their sport–work DC. D1, without skilled work and retired for some time, stated that:

“That spirit you acquired throughout your sports life, plus the one you could have from your origins, is what has helped me to get where I am.”

D1, the oldest athlete in our sample, combined work and sport from a very young age to provide financial support for his family. His socio-historic-cultural context should be taken into account in order to understand that work was the priority and he trained before or after his working day.

In the words of D14, employed in a skilled job:

“My co-workers say it, that in 2 h I do what others do in six, that we have that ability, those acquired principles or abilities.”

##### Underlying Sports Identity

Eleven of the participants developed their DC combining their sports careers with studies that had nothing to do with sports. However, these athletes, after finishing their academic careers, combined work and sport or ended up working after their retirement in companies related to the sports sector—which could be an indicator of the underlying sports identity that we alluded to. They did this either by adapting their studies to work or by putting the skills acquired in higher education at the service of the company. For example, D10 studied journalism and went to a media specialized in sports to be interviewed after becoming world champion. When she was asked what she would like to do after her retirement, she told us the following:

“I said I wanted to work there. They called me the next day and they offered me a collaboration. Then they offered me a part-time collaboration and, after the Athens 2004 Olympic Games, my boss asked me if I was able to keep on doing what I was doing at that moment. I said yes and he told me he was giving me an 8-h contract until I retired, and they kept me there.”

#### Barriers

##### Effort and Requirement Perception

All the athletes in our sample claimed that combining work–sport meant psychological overrun due to the high requirements involved. Entering the labor market may require the development of a new identity as a worker. This difficult process of identity making can be a psychological cost overrun for athletes. As can be inferred from the narrative analysis, working at the same time as belonging to the sporting elite involves an extreme effort. In the words of D16:

“It is really hard because I have combined education, sport and work, and I have joined a company where they work intensively. It has coincided with a year in which we train a lot because of the Olympics and, apart from that, education. If I had to grade the sacrifices I have made this year, it would be 10/10.”

### Psychosocial Level

The psychosocial level refers to the significant people in the athletes' environments who have influenced their careers. The emerging supraordinate category was the “social support perceived,” being understood as the set of behaviors and attitudes developed by the social environments of the athletes, which are likely to facilitate the combination of sport and work.

#### Resources

##### Family Role

In connection with the family support perceived, athletes combining sport and work seem to express its importance. D16 narrates the support and advice received regarding how and/or when to access the labor market:

“I was worried because I was 25 and I hadn't started to work. My mother used to tell me: ‘you have your entire life to work, but it is necessary to start with an internship or as an accountant…” My father told me the same: “you have all your life to work, for sure you have to sacrifice, but also you have to know how to enjoy things.” That is what I have tried, to maintain that balance.”

This example illustrates how the support perceived on the part of the family structure mitigates the feeling of stress due to the late entry into the labor market.

#### Barriers

##### Social Network

The family was excluded from the social network category, which refers to coaches, peers, and friends who, as we have found, seem not to be a facilitating resource for the DC. In the best cases, their role is as neutral agents who do not participate; in the worst ones, they can act as barriers, as can be inferred from the narrative of D4:

“The only memories are that everyone told me I was crazy. Nobody advised me to do elite sport, rather the opposite, all people advised me: ‘don't go crazy, where are you going to go? And your future and your life?’.”

D6, after being asked about someone who supports her who does not belong to her family, answered:

“No, I told my trainers that I didn't know if this was worthwhile. I couldn't go out at the weekends because all was study, training and work. After all, I haven't got time for anything else, so you get overwhelmed.”

#### Academic–Vocational Level

The academic–vocational level refers to the athletes' experiences during their sports careers in relation to attempting to combine them with education and/or a professional occupation. The emerging supraordinate category was “work–sport combination.” This issue includes the subjective perceptions of the athletes in relation to the resources and the barriers that they have found to being able to develop this combination.

#### Resources

##### Coping Strategies for Retirement

The athletes interviewed narrated that the best way to cope with retirement was to have worked previously in the labor field they would have liked to develop in. We should consider that some of the athletes interviewed (four) made their DC combining work and sport, while all the others (14) completed their studies before entering into the labor market or while they were working. In the words of D2:

“I didn't need it, for not letting go of the opportunity. When they made me the proposal I couldn't say no because they weren't going to make it in 2 years anyway.”

This athlete related that, at the high point of her sports career, she was offered a junior position within the labor field she had studied. Despite the previous sacrifice of the DC education–sport, she decided to continue her efforts to combine sport and work as a strategy for the future.

##### Facility for the Combination Working Within the Same Sport

This refers to the athletes who have more opportunities since they develop a DC working in their sport (usually as trainers). In the words of D5:

“The gym was indeed compatible with training. You were in that world and you could live.”

Or in the words of D3:

“I had already been with the club which had been founded for 2 years, teaching from Monday to Thursday. I had to leave the CAR (High-Performance Center) because the training sessions were early. I spent many hours in the classes, earning a lot of money, and when they finished, I went to the gym and trained with my father. I had to choose between the CAR or the classes, and I went to the classes.”

In any case, sport performance was affected. D3 achieved his major sports success after making the decision explained above, becoming world and European champion on several occasions.

##### Facility for the Combination Within the Private Labor Sector

Those athletes who successfully combined work and sport, working in companies related to their field of study or who valued the skills acquired during their higher education, narrate the facility for combining provided by the employers. In the words of D14:

“I said: “yes but you cannot force me to have eight face-to-face hours. With the telephone whatever you want, e-mails whatever you want, but my training sessions are sacred and my study schedule or moments in which I have exams or deliveries, or holidays leave me alone.” I have never had problems. I was given days without wasting holidays.”

In the words of D10:

“I was promoted while I was still competing. I retired with the conditions of that moment, which were: “I come and go whenever I want to; I go on the journeys…”.”

#### Barriers

##### Obstacles in Positions Within Companies Not Dedicated to Sports

This refers to the obstacles that the athletes who develop their DC working within a labor field that is not related to sport could find, with or without having a higher education. In the words of D8:

“Since I hadn't quit the sport, it was hard for me. I was overwhelmed: “I don't eat, I have to train, I can't…,” I didn't get used to it. I was there for a year, but I kept on being more into the sport.”

This athlete tells her story combining sport with an occupation related to her education. Since it is not a company related to the sports field, the facility that the athletes from the previous section had were impossible, making the combination unfeasible.

### Financial Level

The financial level reflects the economic support that the athletes could have from different sources (family, sponsors, grants, etc.) during their DC trajectory. The emerging supraordinate category here was “security perception,” since it is a subjective view of the athletes of the help received from different entities.

#### Resources

##### Income

Given the heterogeneity of the sample, retired, active, professional, amateur, Olympic, and non-Olympic athletes, the economic element is variable. To provide homogeneity to this subordinate category, it seemed appropriate to focus on the active athletes to minimize this variability. Regarding the professional athletes, the income received for practicing their sport is a valuable resource when it comes to facing their sport–work DC since they do it from a position of security. In the words of athlete D11:

“I am one of the privileged few that could pay for my house, I have had my savings and I think I have lived a golden age in terms of my finances, but I was a separate case.”

This athlete is a professional and Olympic medalist, so apart from his salary, he receives financial supports as an Olympic athlete. His DC combining sport and work is driven by his self-interest in the labor market and in finding a way out when he retires.

#### Barriers

##### Incomes

Having fixed incomes (see the case of the professional athletes) provides balance for the athletes. However, the non-professional ones depend on the government grants that, in turn, depend on the results obtained. This causes these athletes to develop their work–sport DC with more anxiety and choosing unskilled jobs in order to be able to continue. D12 says:

“I earn nothing, the only thing I earn is from the national team and, obviously, for paying for petrol; if I want to go out to dinner, to the cinema or whatever, I also need it.”

This athlete, an Olympic team sport medalist, admitted that she receives a grant but with lower amounts than her team members because she has spent less time on the team, the reason why she combines her studies with an unskilled job and the elite sport.

In the case of D13, a non-Olympic athlete:

“The only thing we earn is the medals we win. The Spanish Federation, what we are entitled to: our expenses. There has never been any support in any way. For a gold medal in a world championship, we earn 3,900 euros. If I were Olympic, I would be receiving grants of 60 or 70 thousand euros.”

These athletes develop their sport–work DC almost compulsorily. In some cases, they could have some sponsorship or, hopefully, their families could support them, but as long as their sports careers last, they know that they have to combine if they want to have an opportunity after their retirement.

##### Institution Perception

Most of the athletes of the sample seemed critical of the policies aimed at the work–sport combination since they considered them practically fictional or ineffective. In this subordinate category, we tried to identify, from the athlete's perception, if the official institutions developed some kind of actions in the case that the athletes chose the parallel trajectory of combining work–sport. In the words of D11:

“The Consejo Superior de Deporte (Spanish Sport Council) is not aware that a person at 26 has a child and has to juggle many balls in the air, the child, the work, the training session…”

In the words of D18:

“Well, in general, they do something, but I think it is more politicking, providing visibility: ‘Look how we take care of our athletes!’ but then, behind the camera, bye bye. No, they have never helped me anyway.”

Specifically, 16 athletes criticized institutional policies, considering them fictional or ineffective.

## Discussion

The purpose of this study was to explore the subjective perceptions of the resources and barriers that the employee-athletes have to face in their DC process. It followed the HACM (Wylleman et al., 2013), considered one of the most useful models in this type of research (Stambulova and Wylleman, 2019; Monteiro et al., 2020) as well as the sport career transition model (Stambulova, 2003). The results suggest that the employee-athletes perceive interesting resources like the facility for the combination within the private labor sector or an underlying sports identity and, on the other hand, barriers like obstacles in positions within companies not related to sport or the athletes' perceptions about the stakeholders and the aid received in relation to this issue, when it comes to confronting their DC process, constituting a broad range of phenomenological experiences.

The importance of these findings lies in the fact that we can begin to understand the experience of the employee-athletes. Only in this way will we be able to continue research in the hope of solving the difficulties they have to face, as well as boosting the resources they already have.

More in detail, at the psychological level, it seems that one of the major barriers that the employee-athletes found was the perception of great effort and requirements that they faced. Work pressure, derivative of the inflexibility of the schedules and the responsibility of achieving business objectives (particularly in skilled work), could be the main cause. Certainly, when combining sport and work, the competition schedule, journeys, and training sessions are difficult for employees (athletes), as well as for employers (Debois et al., 2015; Tekavc et al., 2015). Perhaps, being a student-athlete can be easily integrated as part of the life cycle, but the athlete does not conceive working until his or her retirement, so being an employee-athlete after having completed a DC with studies is difficult to integrate. According to the athletes, in your studies, you can “relax, fail in some subject if you do not arrive on time,” but that is something inconceivable and unacceptable in the labor sphere. In respect of the resources, the transference of sports values to the labor sphere was identified, where a set of values coming from sport and embodied in athletes is perceived as a guarantee of success when dealing with work demands (González Fernández and Torregrosa, 2009). In this respect, recognizing the competences their athletic careers have given them allows athletes to capitalize on their transferable skills in a new career and is seen as empowering (Carapinheira et al., 2018). These values are presented by the athletes during the interviews, and these helped them to perform better at work while they continue their sports careers, which is in line with the findings of the European program that has studied the employee-athlete's reality (B-WISER, 2018). This finding can be very interesting and important for employers because of its practical implications. Sometimes, the image of the athletes can be as a person who only knows how to do sport. However, this result suggests that they can be great workers who bring a lot of value to the business world. The resource called “underlying sports identity” was also mentioned. Although high-level sport means that many actions and behaviors of the athletes tend to create a strong sports identity from a young age (Çetinkaya and Yetim, 2015), many of the interviewed athletes developed a DC combining education (not related to the sport they practiced) and sport. This appears to result in a multidimensional identity at the expense of a unidimensional sports identity (Lavalle and Robinson, 2007; Warriner and Lavalle, 2008; Moreno et al., 2020). However, the results suggest that a kind of underlying sports identity exists, and, regardless of the higher education received, that identity linked their education to the sporting sphere. This contributed to finding jobs in which the combination was easier because they also belonged to the sports sphere. The transition to retirement is characterized by maintenance of a strong athletic identity, so the negative consequences of termination could be attenuated with a relocation in sport. This can promote the feeling of integration and competence for the development of a new life (Dimoula et al., 2013; Carapinheira et al., 2018).

At the psychosocial level, the main resource found was the family role, in which the employee-athletes reveal that the family support and certain advice or strategies given facilitate the work–sport combination, in line with the athlete-students (Moreno et al., 2018). The social network, including co-workers, coaches, peers, and friends, could be considered as supportive (Carapinheira et al., 2018) or as a barrier (B-WISER, 2018). In our study, athletes perceived as barriers the social pressure and the lack of support from friends and from the coaches, whom the athletes in this research considered as being only concerned with the sports results.

At the academic–vocational level, the first resource identified was the facilitation of the combination within the private labor sector. The athletes with higher education who have entered the skilled labor market later, while they were still developing their sports careers, narrate that their companies—generally related to sports—supported their DC by reaching beneficial agreements. These results are also in line with the overall perspective that the participating athletes showed in the B-WISER (2018), where, in the structural coordination section, the flexible structure provided by employers is identified as an example of good practice for the combination. This is a very significant fact, but it should be remembered that it refers to the companies within the sporting sphere and athletes with international results, so it may be that they have more advantages than others who did not achieve such results. The other resource identified in the academic–vocational level is labeled coping strategies for retirement. According to Vilanova and Puig (2016), the strategist student-athletes seem to transit better to retirement than the ones who do not develop strategies for doing so. Similarly, the employee-athletes also develop strategies to enter the labor market before they obtain a higher education degree to gain the experience that is necessary to obtain a job after retirement and to be able to go through the transition in a smoother way. This issue needs to be properly assessed, keeping in mind that during the financial recession periods (2008–2018), it was extremely hard for young people to enter the labor market, so they tended to stay longer in the education system (Organisation for Economic Cooperation Development, 2017). A survey conducted in Spain on a sample of retired Olympic athletes showed that 51% of them had studied and 17% had worked during their career high point (Vilanova and Puig, 2013). Although the percentage of employee-athletes shown in this research does not seem to be excessively high, we cannot ignore that the athletes who combined work and sport acquired professional training previously, which would be particularly valued in the labor market (Barriopedro et al., 2018). In any event, this finding is in line with what in B-WISER (2018) has been perceived as a resource called self-regulation, which includes planning, self-evaluation, effort, and the self-efficacy. In this regard, it seems that both managers and athletes need to know how to look at the trends of career development during the mastery stage to prepare the retirement transition and their career out of sport (Monteiro et al., 2020).

With regard to the barriers, it has been identified that the athletes who had temporary, unskilled jobs combined with education and/or sport did not enjoy the same benefits. In general terms, these athletes had applied for government aids or belonged to non-Olympic sports. The athletes who had lost Olympic grants or who were non-Olympians perceived themselves as helpless and neglected, corroborating the fact that the athletes themselves, like society in general, identify being an Olympian as highly prestigious (McEwen et al., 2018). Likewise, it has been verified that if they do not work in a company related to the sporting sphere, the employers' inflexibility for the correct combination was more evident.

At the financial level, given the heterogeneity of the sample, different scenarios may arise, making incomes identifiable either as a resource or as a barrier. As a consequence, athletes who do not receive government aid, the non-Olympic ones, the ones who lose grants because of the results, and the amateurs, are compelled to combine sport and work. The need to earn a living is therefore perceived as a barrier since they are forced to develop a DC even if they do not receive any institutional aid to do so. However, this reality can turn them into more independent and self-sufficient individuals since they can develop skills that could have a positive impact on their lives in general, besides being able to manage DC demands effectively (Aquilina, 2013). On the other hand, there are the athletes with skilled jobs after completing their higher education degree, the Olympic ones with government grants, and the professionals who have considerable incomes, so in this way, it could be considered as a resource.

At this level, a clear barrier was identified in their perceptions of the sports entities, although some studies on this topic reveal this perception of the institutions as a barrier within academia (González Fernández and Torregrosa, 2009; Cosh and Tully, 2014; Stambulova and Wylleman, 2015; Moreno et al., 2020), the current research highlights this problem also for employee-athletes. The lack of perceived support from sport officials, sport organizations, former coaches, and psychologists has been widely reported (Dimoula et al., 2013). The B-WISER (2018) study suggested that athletes perceive policies designed to support employee-athletes as ineffective. Among the reasons proposed, one may find, for example, that the plans are made without considering the athletes since they only focus on the job search or are poorly designed. There is research that underlines that the athletes would like to have such support and reports that support programs should be developed for counseling and awareness regarding their future and to advise them in professional and financial decisions (Carapinheira et al., 2018). Likewise, the subjects of the present research have the perception that the programs or plans are designed by the entities just to show that they are doing something for the athletes.

For all the above, and taking into account that in the critical review of DC research carried out by Stambulova and Wylleman (2019) only two studies out of 42 considered some aspects of a DC in sport and work, we believe that we are increasing knowledge about this topic, as the lack of research is significant on employee-athletes and the literature needs to fill this gap. Therefore, this study seeks to highlight this issue in order to gain an in-depth understanding of it and to be able to act accordingly.

With regard to the limitations of this study, a first consideration should be addressed concerning the possibility to generalize its results (Braun and Clarke, 2006; Smith, 2018). Whether or not the concept of validity may be applicable to qualitative methods (Onwuegbuzie and Leech, 2007), it is important to underline how the quality and size of the sample provide credibility and transferability (Torregrosa et al., 2004). Furthermore, we cannot deny other factors that should be taken into account such as the financial-family situation or the socio-historic-cultural context of the retired athletes. These contexts (e.g., the seventies and eighties of the twentieth century) become important since the government aid was fictitious or insufficient, topics that are hard to ignore in this kind of research (Stambulova and Ryba, 2013; Stambulova et al., 2014).

For future studies, it would be necessary to extend the in-depth investigation to the athletes of our sample who directly chose to work and keep on with their sports careers or who combine work and sport when finishing their education–sport DC. The existing differences between Olympians and non-Olympians, as well as between professionals and amateurs, could be another interesting subject to address. Similarly, it would be interesting to study in-depth the differences between women and men regarding key topics (e.g., salary gap or difficulties for combining work with family). Finally, further research is advisable concerning how to generate strategies and/or policies on behalf of the institutions to help those athletes who, sometimes, come to develop a “triple career” in which they combine education, work, and sport to survive.

## Conclusions

Keeping in mind the lack of studies focused on the DC of the employee-athletes (Stambulova and Wylleman, 2019; Monteiro et al., 2020), this research attempts to deepen the understanding of the Spanish elite athletes' experiences in relation to combining work and sport. The results obtained highlight the resources and barriers these employee-athletes face during their DC, providing a more global vision for future interventions with this population. Taking their reality into account, it has been confirmed that they have a valuable resource in the transference of sports values to the labor sphere, giving them the possibility of being highly valued employees due to their efficiency and commitment. Perhaps, support from official institutions for employers could be considered to facilitate the DC of athletes combining work and sport. This could be an opportunity for employers to see the added value that the athletes can bring to their companies. Family support or the facility that certain companies, related to the sporting sphere, bring for combining work and sport is also important. In this respect, it is emphasized that the perception of being forgotten by the institutions gives them a certain feeling of helplessness. This feeling seems to suggest that maybe the question should be raised with government agencies and their actions or programs again, in line with one of the findings of an IOC project that indicated the need for a line of employment promotion for former athletes (López de Subijana et al., 2016). Having collated the results obtained in this research with the ones obtained in the project B-WISER (2018), it has been found that they coincide. This seems to suggest that it would be necessary to study, in detail, how the reality of these employee-athletes can be addressed to facilitate both their DC process and their subsequent socio-labor integration after their retirement.

## Data Availability Statement

The original contributions presented in the study are included in the article/supplementary material, further inquiries can be directed to the corresponding author.

## Ethics Statement

The studies involving human participants were reviewed and approved by Comisión de investigación Universidad Europea de Madrid. The patients/participants provided their written informed consent to participate in this study. Written informed consent was obtained from the individual(s) for the publication of any potentially identifiable images or data included in this article.

## Author Contributions

RM contributed to this work in these tasks: conceptualization, carrying out interviews, data curation, formal analysis, investigation, methodology, writing–original draft, and writing–review and editing. JC contributed to this work in these tasks: conceptualization, formal analysis, investigation, methodology, writing–original draft, and writing–review and editing. CL contributed to this work in these tasks: formal analysis, investigation, methodology, visualization, and writing–review and editing. All authors contributed to the article and approved the submitted version.

## Conflict of Interest

The authors declare that the research was conducted in the absence of any commercial or financial relationships that could be construed as a potential conflict of interest.
